# Prognosis of Pregnancy-Associated Breast Cancer: A Systematic Review of Contemporary Observational Studies

**DOI:** 10.3390/medsci14010120

**Published:** 2026-03-03

**Authors:** Dimitrios Zouzoulas, Tilemachos Karalis, Iliana Sofianou, Panagiotis Tzitzis, Themistoklis Mikos, Eleni Timotheadou, Grigoris Grimbizis, Dimitrios Tsolakidis

**Affiliations:** 1 1st Department of Obstetrics & Gynecology, Aristotle University of Thessaloniki, “Papageorgiou” Hospital, 56403 Thessaloniki, Greece; 2 Department of Oncology, Aristotle University of Thessaloniki, “Papageorgiou” Hospital, 56403 Thessaloniki, Greece

**Keywords:** pregnancy-associated breast cancer, postpartum breast cancer, prognosis, breast neoplasms, survival outcomes

## Abstract

Background/Objectives: Pregnancy-associated breast cancer (PABC) is uncommon but increasingly encountered as more women delay childbearing. Its prognostic impact remains controversial, particularly for cancers diagnosed in the early postpartum period. We aimed to synthesize contemporary evidence on the prognosis of breast cancer diagnosed during pregnancy or within 12 months after delivery compared with breast cancer in other young women, with a specific focus on differences between pregnancy time and postpartum disease. Methods: We performed a systematic review of observational studies published from 2005 onwards that reported oncologic outcomes for women with invasive PABC versus non-PABC comparators or PABC-only cohorts with internal timing comparisons. PubMed, Cochrane Library, Scopus and ClinicalTrials.gov were systematically searched using predefined strategies. Two reviewers independently screened records, extracted data and assessed risk of bias using the ROBINS-E tool, treating PABC status as the exposure. Because of substantial heterogeneity in PABC definitions, outcomes and adjustment sets, no meta-analysis was performed. Results: Twenty-one observational studies (single-center, multicenter and population-based) were included. PABC cases more often presented with larger tumors, higher nodal burden, high-grade and hormone receptor-negative/HER2-positive phenotypes and worse survival compared to non-PABC controls. In most contemporary cohorts that delivered guideline-oriented therapy and adjusted for stage and tumor biology, a diagnosis during pregnancy was not an independent predictor of poorer disease-free or overall survival. In contrast, multiple large registry and institutional studies reported significantly higher risks of recurrence and death for cancers diagnosed in the early postpartum period, even after multivariable adjustment. Conclusions: Current evidence suggests that pregnancy itself does not inevitably worsen breast cancer prognosis when treatment is not compromised. However, breast cancers diagnosed soon after childbirth represent a distinct high-risk subgroup. These findings support full-intensity, guideline-based therapy during pregnancy and highlight the need for special attention and further research focused on postpartum breast cancer.

## 1. Introduction

### 1.1. Rationale

Pregnancy-associated breast cancer (PABC) is usually defined as an invasive breast cancer (BC) diagnosis during pregnancy or within the first year after delivery [1,2]. Historically, pregnancy-associated breast cancer (PABC) was defined as breast cancer diagnosed during pregnancy or within the early postpartum period, sometimes extending up to several years after delivery; more recently, a distinction has been made between pregnancy-time breast cancer (PrBC), diagnosed during pregnancy, and postpartum breast cancer (PPBC), diagnosed within a postpartum window of up to 5–10 years, reflecting differences in tumor biology and prognosis [3]. Although uncommon in absolute terms, it is one of the most frequent malignancies encountered in pregnancy, with an estimated incidence of about 1 in 3000 pregnancies and a rising trend over recent decades [2,4]. This increase appears to be driven largely by the deferral of childbearing into the fourth decade of life, which is a time period during which breast cancer incidence rises. This fact shifted medical attention to whether breast cancer during pregnancy or in the immediate postpartum period carries a distinct prognosis compared with breast cancer in other young women [4].

Clinically, PABC is challenging. Physiological changes in breast size, density and nodularity during pregnancy or lactation can mask palpable lesions and reduce the sensitivity of mammography. These can lead to diagnostic delays and a higher likelihood of large, node-positive, high-grade tumors at the time of diagnosis [1]. Several series have reported an over-representation of biologically aggressive phenotypes, including hormone receptor-negative, HER2-positive subtypes and high Ki-67 percentages among women with PABC compared with age-matched non-pregnant controls [5]. At the same time, concern for fetal safety constrains the administration and the timing of systemic therapies, particularly in the first trimester, requiring careful coordination of surgery, chemotherapy and radiotherapy between oncology and obstetric teams so that maternal treatment is not unduly compromised [5].

Over the last two decades, the long-held assumption that pregnancy itself inevitably worsens prognosis has been questioned. Contemporary multicenter cohorts and guideline-driven series suggest that, when standard therapy is delivered without major modification, women diagnosed during pregnancy can achieve outcomes broadly comparable to non-pregnant women of similar age and stage [6]. In contrast, an accumulating body of population-based and institutional data indicates that breast cancer diagnosed in the early postpartum period may behave differently. Meta-analyses and large cohort studies have reported an excess risk of distant recurrence and death among women diagnosed within several years after childbirth, even after adjustment for stage and tumor biology, raising the question of whether the involuting postpartum breast microenvironment promotes tumor progression [6,7,8,9].

The interpretation of this literature is difficult due to heterogeneity in how PABC is defined and analyzed. Some studies restrict inclusion to breast cancer diagnosed during gestation; others combine pregnancy and postpartum cases into a single “PABC” category; still, others extend the definition to include diagnoses up to five or even six years after delivery [5,6,7]. The extent of adjustment for confounders, the availability of contemporary systemic therapy and the reporting of separate outcomes for pregnancy versus postpartum cases also vary widely between studies. Previous meta-analyses have therefore pooled data across disparate definitions, eras and treatment contexts, making it difficult to draw robust, practice-relevant conclusions [6,7].

Based on this background, there is a clear need for a focused, methodologically transparent synthesis of recent data that uses a clinically meaningful definition of PABC and explicitly distinguishes cancers diagnosed during pregnancy from those arising in the early postpartum period. Such a synthesis is essential in order to provide realistic prognostic information to young women facing breast cancer in the context of pregnancy or immediately postpartum and to guide clinicians in counselling, treatment planning and follow-up.

### 1.2. Objectives

The primary objective of this systematic review was to critically appraise and synthesize contemporary evidence on the prognostic impact of pregnancy-associated breast cancer, defined as invasive breast cancer diagnosed during pregnancy or within 12 months postpartum. Specifically, we aimed to compare disease-free and overall survival in women with PABC compared to non-PABC controls, while considering the influence of tumor stage, biological subtype and adjuvant treatment.

A secondary objective was to explore how differences in the definition of PABC, particularly the postpartum time interval from delivery and the diagnosis of breast cancer during pregnancy and postpartum, affect survival outcomes. Specifically, we aimed to examine whether breast cancer during pregnancy or immediately postpartum displays a distinct prognostic profile. Finally, by systematically mapping gaps, inconsistencies and sources of bias in the available literature, this review seeks to identify priorities for future research and to provide clinicians with a balanced, evidence-based framework for counselling and management of young women confronted with breast cancer during pregnancy or immediately postpartum.

## 2. Materials and Methods

### 2.1. Eligibility Criteria

This study was designed as a systematic review of observational studies, conducted and reported in accordance with PRISMA 2020 recommendations (PROSPERO ID: 1322658). Eligible studies were those that evaluated survival outcomes in women with pregnancy-associated breast cancer and included a comparison with non-pregnancy-associated breast cancer patients. For the purposes of this review, pregnancy-associated breast cancer (PABC) was defined as invasive breast tumors diagnosed either during pregnancy or immediately postpartum, with a primary focus on studies using a cut-off of 12 months from delivery. Studies that adopted slightly broader definitions, such as postpartum windows up to 24 months, were considered on a case-by-case basis and were included in the core analysis only if their findings reflected early postpartum disease.

We considered original observational designs, retrospective or prospective cohorts, case–control studies, and population-based registry analyses. To be eligible, studies had to report time-to-event survival outcomes such as overall survival, disease-free survival, event-free survival, recurrence-free survival or breast cancer-specific survival. Studies limited to ductal carcinoma in situ, male breast cancer or exclusively metastatic (stage IV) populations were excluded. Mixed-stage cohorts were eligible if early-stage disease predominated and survival analyses were not restricted to metastatic cases.

Comparative data were required. Studies had to include either an explicit non-PABC comparison group or adjusted analyses in which pregnancy status or time since childbirth was entered as an exposure variable. Case reports, very small case series with fewer than ten PABC cases, conference abstracts without sufficient methodological detail and purely molecular or pathological studies without survival data were excluded. Systematic reviews, meta-analyses and narrative reviews were not treated as primary evidence but were examined to identify additional original cohorts and to contextualize findings.

We restricted inclusion to articles published in peer-reviewed journals between 1 January 2005 and the date of the final search (1 December 2025) in the English language, involving only human female participants. When multiple publications reported overlapping data from the same cohort, we retained the most comprehensive report to avoid duplication.

### 2.2. Information Sources

A systematic search of electronic databases was performed to identify relevant studies. The following databases were interrogated: MEDLINE via PubMed, Cochrane Library (CENTRAL) and Scopus search engine. To minimize the risk of missing eligible studies, we additionally searched ClinicalTrials.gov for ongoing or completed observational cohorts and interventional studies that might not yet have been fully published.

The database search was supplemented by manual strategies. Reference lists of all included articles and of systematic or narrative reviews and meta-analyses on PABC were screened for additional eligible studies. Grey literature, such as dissertations and non-indexed conference abstracts, was not systematically searched because of the focus on fully peer-reviewed survival outcomes and to ensure sufficient methodological detail for quality assessment.

### 2.3. Search Strategy

Search strategies were tailored to each database but shared a common conceptual framework. The search combined terms referring to breast cancer, pregnancy, the postpartum period, prognosis and survival. In PubMed, the strategy included both MeSH terms and free-text keywords. The PubMed search string was as follows:

(“pregnancy-associated breast cancer” [tiab] OR “pregnancy associated breast cancer” [tiab] OR “breast cancer during pregnancy” [tiab] OR “breast cancer in pregnancy” [tiab] OR “breast cancer and pregnancy” [tiab] OR “postpartum breast cancer” [tiab] OR “postpartum breast cancer” [tiab] OR “lactation-associated breast cancer” [tiab] OR “lactation associated breast cancer” [tiab] OR ((“Breast Neoplasms” [Mesh] OR breast cancer [tiab] OR breast neoplasm* [tiab]) AND (pregnancy [tiab] OR pregnant [tiab] OR gestation [tiab] OR postpartum [tiab] OR “postpartum” [tiab] OR puerperium [tiab] OR peripartum [tiab] OR lactation [tiab]))) AND (“prognosis” [Mesh] OR prognosis [tiab] OR survival [tiab] OR “overall survival” [tiab] OR “disease-free survival” [tiab] OR mortality [tiab] OR outcome* [tiab] OR “hazard ratio” [tiab]).

In Scopus, searches were conducted using the TITLE-ABS-KEY fields and applying equivalent Boolean logic. The Cochrane Library search was limited to trials and controlled observational studies using simpler keyword strings for breast cancer and pregnancy. Clinicaltrials.gov was searched for breast cancer as the condition/disease, female participants and observational studies from 2005 onwards.

In all databases, filters were applied for human-only studies, female sex, peer-reviewed articles and the predefined time period from 2005 onwards.

### 2.4. Selection Process

All records identified through database and registry searches were imported into a reference management system, where duplicates were removed. The selection process followed a two-stage approach.

In the first stage, titles and abstracts were screened independently by two reviewers to determine potential relevance. Articles were excluded at this stage if it was clear that they did not involve breast cancer patients, did not relate to pregnancy or the postpartum period, were not original studies or did not report survival outcomes. Records for which eligibility remained uncertain were carried forward to full-text review.

In the second stage, full texts of potentially eligible articles were obtained and assessed independently by the same two reviewers using the inclusion and exclusion criteria of this systematic review. Particular attention was paid to the authors’ definition of PABC, the presence of the non-PABC comparator group and the availability of time-to-event data. Discrepancies between reviewers regarding study eligibility were resolved through discussion and, when needed, consultation with a third senior reviewer.

### 2.5. Data Collection Process

For each study that met the eligibility criteria, data were extracted independently by the two reviewers using a standardized data collection form on Microsoft Excel software [16.105.1 (26011816)]. The form was designed to capture key methodological and clinical information relevant to prognosis in PABC and to facilitate consistent comparison across heterogeneous study designs.

Data extraction was performed directly from the published articles and any available online supplementary materials. Contact with study authors was considered when critical data were missing or hard to interpret, although most included studies provided sufficient detail. After independent extraction, the two forms for each study were compared, and any discrepancies were resolved by consensus, with arbitration by a third reviewer if needed.

### 2.6. Data Items

The data collected from each study included patient characteristics for both the exposure and comparator groups, adjuvant treatment and survival outcomes. Specifically, we recorded the first author, year of publication, country, type of institution or registry, study design (retrospective or prospective cohort, case–control, registry-based) and recruitment period.

Patient characteristics included the number of women with PABC and, in the comparator group, age at diagnosis; disease stage; histologic type; tumor grade; and, when available, molecular subtype (estrogen receptor, progesterone receptor, HER2, Ki-67). The timing of PABC diagnosis was carefully documented. A categorization between breast cancer during pregnancy, during lactation and within a specified postpartum time period was made, as well as whether pregnancy-only and postpartum cases were analyzed together or separately.

Data from the control group included information about the parity of women (nulliparous women, parous women with remote pregnancies or a mixture of non-pregnant patients). Further information about whether matching was employed based on age, stage, year of diagnosis or center was collected.

For survival outcomes, we recorded data on overall, disease-free and breast cancer-specific survival, as well as local or distant recurrence.

### 2.7. Study Risk of Bias Assessment

Risk of bias in individual studies was assessed using the ROBINS-E tool [10], which is specifically designed for non-randomized studies evaluating the effect of an exposure on an outcome. In this review, the exposure was pregnancy-associated status for breast cancer (PABC vs. non-PABC). Two reviewers independently rated each study across seven domains: bias due to confounding, classification of the exposure, selection of participants, post-exposure interventions (differences in treatment), missing data, outcome measurement and selection of the reported result.

Judgments for each domain were made using the ROBINS-E questions and categorized as low risk, some concerns, high risk or very high risk. Attention was paid to the accuracy of pregnancy and postpartum timing and to how confounders (age, stage, nodal status, tumor biology, treatment) were addressed. For matched case–control studies, the tool was applied by analogy, treating selection of cases and controls as selection into the exposed and unexposed groups. Disagreements between reviewers were resolved by discussion.

### 2.8. Effect Measures

The primary effect measures of interest were hazard ratios for overall survival and disease-free or event-free survival comparing women with PABC to either non-pregnant or remote postpartum breast cancer patients. When hazard ratios were not reported, we noted alternative measures such as relative risks or odds.

Given the expected clinical and methodological heterogeneity, we did not plan a single pooled summary effect. Instead, adjusted hazard ratios from multivariable analyses were prioritized in the narrative synthesis with attention to which confounders were included and how strongly pregnancy or postpartum status remained associated with prognosis after adjustment.

### 2.9. Synthesis Methods

Because of substantial heterogeneity in definitions of PABC, postpartum cut-offs, staging, treatment approaches and survival outcomes reporting, a narrative synthesis was chosen as the method of data management. Studies were first grouped according to their PABC definition and clinical context: those restricted to breast cancer diagnosed during pregnancy, those including both pregnancy and immediately postpartum (up to 12 months from delivery) as a single PABC group and those that provided separate analyses for postpartum breast cancer diagnosis before and after the first year from delivery.

Special attention was given to assessing if the adverse prognosis of breast cancer during pregnancy or postpartum persisted after controlling for stage, nodal involvement, grade, molecular subtype and treatment. When multiple studies addressed similar populations and definitions, we explored the consistency of findings qualitatively.

### 2.10. Reporting Bias Assessment

Reporting bias, including publication bias and selective outcome reporting, was assessed qualitatively. Formal statistical methods such as funnel plots were not applied, as no meta-analysis was performed, and the number of relatively homogeneous studies per subgroup was limited. Instead, we examined whether small single-center series and larger population-based registries tended to systematically report different directions or strengths of association.

### 2.11. Certainty Assessment

We planned to assess the certainty of evidence for each outcome using the Grading of Recommendations Assessment, Development and Evaluation (GRADE) approach. This tool evaluates domains including risk of bias, inconsistency, indirectness, imprecision and publication bias. It classifies the overall certainty of evidence as high, moderate, low or very low. Two reviewers independently conducted the GRADE assessments, and any discrepancies were resolved by discussion or, if needed, consultation with a third reviewer.

## 3. Results

### 3.1. Study Selection

The literature search across the predefined sources yielded a total of 4748 records from the following databases: PubMed (2179 records), Cochrane Library (545 records), Scopus (620 records) and 1404 records identified through ClinicalTrials.gov. After the initial removal of duplicate entries (571 records) and the exclusion of citations that did not meet basic eligibility criteria (non-English language, non-human studies or predominantly male populations; 221 records), 3956 unique records remained for title and abstract screening.

Of these 3956 records, 3173 were excluded at the preliminary screening stage because they clearly did not fulfill the inclusion criteria. The remaining 783 articles were retrieved for full-text review and assessed in detail for relevance and eligibility according to the predefined criteria. This full-text evaluation led to the exclusion of 762 additional studies for reasons such as inappropriate definition of pregnancy-associated breast cancer, absence of survival outcomes or lack of a suitable comparator group. Ultimately, 21 studies were judged to meet all criteria and were included in the present systematic review: Ali et al. (2012) [11], Amant et al. (2013) [12], Bae et al. (2018) [13], Baulies et al. (2015) [14], Boudy et al. (2018) [15], Choi et al. (2019) [16], Dimitrakakis et al. (2013) [17], Genin et al. (2016) [18], Gwak et al. (2022) [19], Han et al. (2020) [20], Johansson et al. (2018) [21], Jo et al. (2022) [22], Kim et al. (2017) [23], Madaras et al. (2014) [24], Martín Cameán et al. (2024) [25], Moreira et al. (2010) [26], Murphy et al. (2012) [27], Ploquin et al. (2018) [28], Waheed et al. (2025) [29], Zhang et al. (2021) [30] and Zhang et al. (2023) [31].

**Figure 1:**
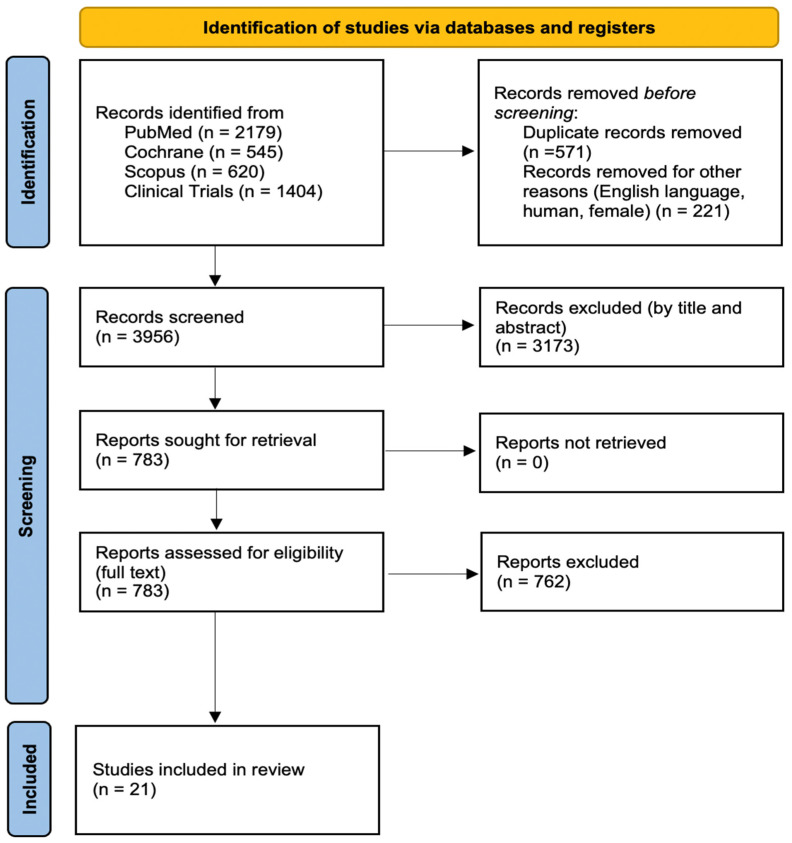
PRISMA flowchart.

This systematic review follows the PRISMA guidelines [32], and the detailed flowchart summarizing this selection process is presented in Figure 1.

### 3.2. Study Characteristics

The final evidence base consisted of 21 observational studies enrolling women with pregnancy-associated breast cancer and a comparison group of non-pregnancy-associated breast cancer in most cases, with the exception of one single-arm contemporary cohort. All studies were published between 2005 and 2025, while the underlying diagnostic periods extended from the mid-1980s to the late 2010s. Designs included classic matched case–control series, institutional or national cohorts and large population- or registry-based datasets. No randomized studies were identified. Most cohorts restricted inclusion to women aged ≤45–50 years at diagnosis and stage I–III disease, while a minority also included de novo stage IV presentations.

Across studies, the PABC definition was broadly similar but not identical. The majority defined PABC as breast cancer diagnosed during pregnancy or within 12 months after delivery (Moreira et al. [26], Ali et al. [11], Murphy et al. [27], Madaras et al. [24], Dimitrakakis et al. [17], Baulies et al. [14], Genin et al. [18], Kim et al. [23], Bae et al. [13], Zhang et al. [30], Zhang et al. [31], Gwak et al. [19], Jo et al. [22], Martín-Cameán et al. [25] and several Chinese series [20,23]). A smaller group limited their analyses to cancers diagnosed during an ongoing pregnancy, without postpartum cases (Amant et al. [12], Boudy et al. [15], Ploquin et al. [28]), whereas the large Swedish register study by Johansson et al. extended the postpartum window to 24 months after childbirth. Han et al.’s [20] study included patients from the Chinese registry cohort and focused on biologic subtype distribution in 203 PABC cases compared with several tens of thousands of non-PABC tumors, using a definition that encompassed both pregnancy and immediate postpartum breast cancer. The single-arm Pakistani series by Waheed et al. [29] included women diagnosed during pregnancy or in the first postpartum year but did not recruit a non-PABC comparison group. This heterogeneity in temporal definitions of “pregnancy associated” was a key source of between-study variability.

A first cluster of studies consisted of relatively small, single-center or regional matched case–control series designed to compare clinicopathologic features and survival between PABC and non-PABC women treated in the same institutions. Moreira et al.’s [26] paired case–control study from Brazil included 87 women with PABC and 252 controls matched on age and year of diagnosis, all treated in two oncology centers. Ali et al. [11], in a retrospective study, identified 40 women with PABC (17 during pregnancy, 23 postpartum) and 40 non-pregnant controls matched for age and stage. Murphy et al. reported on 99 women with current or recent pregnancy-associated breast cancer managed at a USA cancer center, using a 2:1 matching strategy that yielded 198 non-PABC comparators. Three European case–control series, from Madaras et al. [24], Dimitrakakis et al. [17] and Martín-Cameán et al. [25], contributed 31, 39 and 34 PABC patients, respectively, each compared with one or more non-PABC controls selected on age, stage and year of diagnosis. Furthermore, Baulies et al. [14] included 56 PABC and 73 non-PABC cases, while Genin et al. [18] analyzed 87 PABC cases versus 174 matched controls treated in a comprehensive cancer center, focusing particularly on local regional recurrence. Two more recent Chinese matched series (Zhang et al. [30] and Zhang et al. [31]) included 41 and 40 PABC patients, respectively, each matched to one or two non-PABC controls by age, stage and year of diagnosis. Follow-up in these case–control studies generally ranged from about 3 to >6 years, providing data on disease-free and overall survival.

A second group of studies was multicenter or network-based hospital cohorts from Europe and Asia that combined breast cancer during pregnancy and postpartum, but with varying emphasis. Amant et al. conducted an international registry study for breast cancer during pregnancy, including 447 women with breast cancer during pregnancy, of whom 311 met strict eligibility criteria and were compared with 865 non-pregnant controls with stage I–III disease. Two French studies included women from the national CALG network and multiple oncology centers. Boudy et al. [15] assembled 81 women with breast cancer during pregnancy and 104 non-PABC controls managed at a national expert center, using propensity score methods to address baseline imbalances. Ploquin et al. [28] conducted a case–control study from 27 French centers and enrolled 111 women with breast cancer during pregnancy and 253 matched non-pregnant controls with early-stage disease, with a primary endpoint being 5-year overall survival. Kim et al. [23] analyzed 344 PABC cases from the Korean Breast Cancer Society registry and compared them with a larger set of matched non-PABC controls, while Bae et al. [13] used the same registry to explore intrinsic subtypes and prognosis in 411 women with PABC against a background cohort of more than 83,000 young breast cancer patients. Choi et al.’s [16] study from Korea also contributed 18 women diagnosed with breast cancer during pregnancy and 45 women up to 12 months postpartum, compared with a much larger national database.

Large population-based or registry studies provided complementary information on incidence patterns, tumor biology and long-term outcomes. Johansson et al. used the Swedish national registry to identify 778 women with breast cancer during pregnancy or within 24 months after delivery and compared them with 1661 parous women with breast cancer not associated with pregnancy and an additional nulliparous reference group. Moreover, Han et al. [20] conducted a Chinese registry study, including 203 PABC cases, and compared their molecular subtype distribution with more than 43,000 non-PABC cases diagnosed in the same institution. Gwak et al. [19] used data from the Korean registry to perform a propensity score matching of 410 PABC cases to 1640 non-PABC controls in a 1:4 ratio, with the primary objective being the survival outcomes in the era of modern systemic therapy. On the other hand, Jo et al. conducted the only prospective cohort study. Among 1492 young Korean women enrolled, 1364 were finally analyzed, including 93 with PABC and over 1200 non-PABC patients followed longitudinally.

Finally, two recent series added information from different geographic settings and clinical contexts. Martín-Cameán et al. analyzed 34 PABC cases and 55 non-PABC controls treated in a Spanish tertiary breast unit, with careful reporting of obstetric outcomes in addition to oncological outcomes. Waheed et al. reported outcomes of 44 women with PABC treated in a tertiary cancer hospital in Pakistan. Although no internal non-PABC comparator was included, this series provides contemporary data on staging, treatment patterns and early survival in a high-burden, but resource-constrained environment.

Despite this broad range of designs, several baseline characteristics were consistent across studies. Women with PABC were typically in their early to mid-thirties at diagnosis, younger than most non-PABC comparators. Tumors in the PABC groups were larger, higher grade and with node-positive disease at diagnosis compared to non-PABC patients. Multiple case–control and registry studies reported a higher proportion of hormone receptor-negative and HER2-positive or triple-negative subtypes among PABC, with a higher percentage of luminal B phenotypes compared to age-matched non-PABC controls. Treatment approaches were broadly similar between groups; most women with non-metastatic disease received surgery and anthracycline-based chemotherapy, often with taxanes in more recent cohorts. However, systemic therapy was frequently postponed to the postpartum period in older series and early registry data. Reporting of obstetric and neonatal outcomes varied and were mainly reported in the retrospective cohorts, including only breast cancer during pregnancy cases (Amant et al. [12], Boudy et al. [15], Ploquin et al. [28] and Waheed et al. [29]), and in the prospective study by Jo et al. [22].

Overall, the included studies were heterogeneous in geography, calendar period, PABC definition, design and outcome reporting. This diversity, together with overlapping patient populations in some registry analyses, precludes straightforward pooling of effect estimates. However, they provided a rich descriptive dataset for a narrative synthesis of how pregnancy timing, tumor biology and treatment impact breast cancer survival outcomes in this setting. Each individual study characteristics included in this systematic review is presented in Table 1.

### 3.3. Risk of Bias in Studies

The risk of bias of the included studies was assessed using the ROBINS-E tool, treating pregnancy-associated status (PABC vs. non-PABC) as the exposure of interest. Overall, none of the 21 studies was judged to be at low risk of bias across all domains. Two studies were rated at very high overall risk of bias, eight at high risk, and the remaining eleven at some concerns.

Bias due to confounding (D1) was the most frequent and influential concern. Older single-center case–control series, such as Moreira et al. [26], Ali et al. [11], Baulies et al. [14], Dimitrakakis et al. [17], Genin et al. [18], Madaras et al. [24] and Martín-Cameán et al. [25], only adjusted for a limited set of variables (typically age and stage) and either did not account for tumor biology, treatment differences or time period. Some studies did it only partially, leading to ratings of high or very high risk of confounding. In contrast, large registry-based cohorts and propensity-matched analyses (Kim et al. [23], Bae et al. [13], Gwak et al. [19], Johansson et al. [21], Jo et al. [22], Amant et al. [12] and Ploquin et al. [28]) generally adjusted for a broader set of prognostic factors, including nodal status, grade and receptor status. Therefore, they were judged to have “some concerns” rather than high risk in this domain. Han et al. and Waheed et al. included only PABC and did not formally adjust for confounders, which contributed to their high or very high overall risk of bias judgment.

Bias in the measurement and classification of the exposure (D2) was typically low. In most studies, pregnancy status and timing of diagnosis relative to delivery were obtained from medical records, obstetric databases or national birth registries, which are unlikely to misclassify the presence of pregnancy. However, there were “some concerns” in a minority of series where the postpartum window was not precisely specified or where pregnancy and lactation were not clearly distinguished from more remote postpartum time periods. This raised the possibility of misclassification at the margins of the exposure definition. Primarily, this concern was for some older institutional cohorts and registry analyses with broader postpartum categories.

In terms of selection of participants (D3), several institutional case–control studies sampled patients from tertiary referral centers and matched PABC cases to a convenience sample of non-PABC controls. Because women referred to high-volume centers may differ systematically from those treated elsewhere, and because matching strategies were not always fully described, these studies were rated as having some concerns or high risk in D3. Population-based registries (Johansson et al., Bae et al., Kim et al. and Gwak et al.) had a lower risk of selection bias, as they attempted to include all eligible cases within a defined time period. Han et al. and Waheed et al., who reported only women with PABC in a single hospital, were again considered at high risk due to the possibility of referral and severity bias.

With respect to post-exposure interventions (D4), nearly all studies documented differences in treatment between PABC and non-PABC groups, particularly in older cohorts in which chemotherapy was more often omitted or delayed during pregnancy. Few studies explicitly modelled treatment patterns as time-dependent covariates or evaluated whether undertreatment mediated the association between PABC and survival. As a result, most studies were judged as having some concerns in D4, and those with substantial documented undertreatment and limited adjustment (e.g., older single-center series and PABC-only cohorts) were rated high risk. More contemporary multicenter and registry studies, which reported broadly similar guideline-concordant treatment between groups, were judged to have lower concern in this domain.

Bias due to missing data (D5) was generally low to moderate. National cancer and birth registries provided near-complete follow-up data for survival outcomes, and loss to follow-up was minimal. However, missingness in key covariates, such as receptor status or grade, was common in older retrospective series, and handling of missing data was rarely described in detail. Studies that were missing important prognostic variables for a substantial proportion of patients that were not addressed analytically were judged as “some concerns”. Only a small number of single-center cohorts with poorly reported follow-up or substantial attrition were rated at high risk in this domain.

Outcome measurement (D6) was considered to be at low risk of bias in most studies. Overall survival and disease-free survival were calculated from hospital records, follow-up clinics or national registries, with no evidence that ascertainment differed by pregnancy status. A few older series provided limited information on how recurrences were captured, but there was no indication that detection bias systematically favored one group over another, so these were generally judged as “low” or “some concerns” rather than high risk.

Finally, bias in the selection of the reported result (D7) was mostly judged as “some concerns”. None of the observational studies had pre-registered protocols, and several presented multiple, partially overlapping analyses without clearly specifying which model was the primary. Selective emphasis on crude versus adjusted results or on particular subgroup analyses could not be excluded. However, most studies reported the main survival outcomes (overall and disease-free survival) that would be expected from their design, and there was no strong evidence of extreme selective reporting. This resulted in very high concern, which is uncommon in this domain.

**Figure 2:**
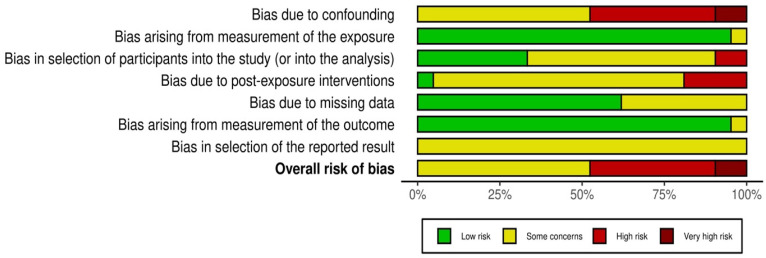
Risk of Bias Summary Plot.

**Figure 3:**
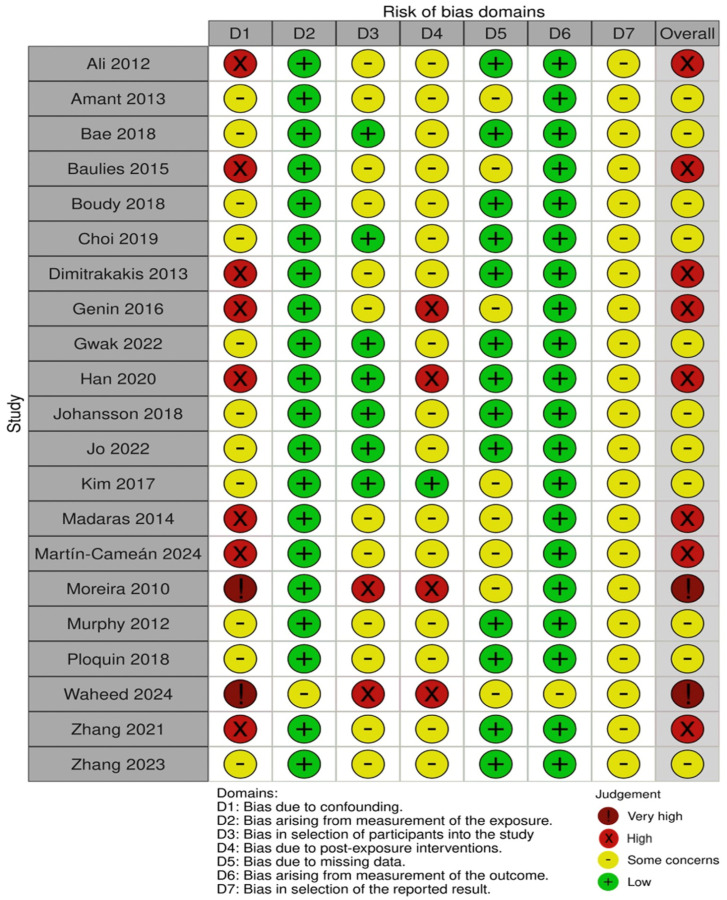
Risk of bias traffic light plot.

In summary, the body of evidence is dominated by observational studies with at least some risk of bias, mainly due to residual confounding and, for smaller institutional series, potential selection bias and unmeasured differences in treatment. Larger, contemporary registry-based and multicenter matched cohorts provide more robust estimates with lower risk of bias but still fall short of the standard of randomized or fully controlled designs. These limitations were taken into account when interpreting the consistency of findings across studies and in grading the certainty of the evidence for the association between pregnancy-associated status and breast cancer prognosis. The above are summarized in Figure 2 and Figure 3.

### 3.4. Results of Individual Studies

The included studies were heterogeneous with respect to design, data source, calendar period, definition of PABC and statistical adjustment. Nevertheless, several consistent patterns emerged. In most series, women with PABC presented with more advanced and biologically aggressive disease than their non-PABC counterparts, leading to worse crude survival. However, in the majority of comparative analyses, the excess risk associated with PABC was minimized, and often disappeared, after adjustment for stage, nodal status and tumor biology. A second recurring observation was that breast cancers diagnosed immediately postpartum tended to have a worse prognosis compared to those diagnosed during pregnancy, suggesting that the timing of diagnosis in the immediate postpartum time period might be clinically relevant. These results are summarized in Table 2.

#### 3.4.1. Institutional Case–Control Cohorts

Several single- and multicenter matched case–control studies compared women with PABC to non-PABC controls treated in the same institution. Moreira et al., in one of the earlier series, paired 87 PABC cases with 252 premenopausal controls. Women with PABC had larger tumors, higher nodal positivity and their unadjusted survival was inferior. However, once the analysis controlled for nodal status and stage, pregnancy-associated status was no longer an independent predictor of disease-free and overall survival. The authors attributed the crude survival disadvantage largely to delayed diagnosis.

Ali et al. reported similar findings in a cohort of 40 women with breast cancer during pregnancy or ≤1 year postpartum, each matched to a non-pregnant control by age and stage. PABC cases exhibited higher grade and more frequent hormone receptor negativity, but matched analyses and multivariable Cox models showed no significant difference in disease-free and overall survival between groups. Dimitrakakis et al. and Madaras et al., using comparable case–control designs, also observed more advanced stage at diagnosis among PABC cases, but did not find pregnancy status to have an independent adverse effect on prognosis after matching for key clinicopathologic variables.

Baulies et al. analyzed 56 PABC cases and 73 matched controls. PABC tumors were more proliferative and of higher grade, with a higher frequency of lymphovascular space invasion. Crude survival was numerically worse in the PABC group, yet differences in disease-free and overall survival did not reach statistical significance once age, stage and grade were taken into account. Recently, Martín-Cameán et al. evaluated 34 PABC cases and 55 non-PABC controls. Again, PABC cases had larger, higher-grade and more often hormone receptor-negative tumors, with a worse unadjusted survival. However, after multivariable analysis, pregnancy-associated status itself was not an independent predictor for survival.

Two Chinese matched case–control studies, by Zhang et al., provide additional contemporary data. In both the 2021 and 2023 series, PABC cases (defined as cancers diagnosed during pregnancy or ≤1 year postpartum) were matched to non-PABC controls on age, stage and year of diagnosis, with some partial matching on subtype. Before matching, PABC cases more often had nodal involvement and lymphovascular space invasion, but after matching, no significant differences in disease-free or overall survival were seen. Together, these institutional studies point to a consistent pattern: PABC is associated with more adverse baseline features and worse crude outcomes, but once patients are compared on equal footing, pregnancy itself does not appear to confer a strong additional prognostic decline.

An exception within this group is the study by Genin et al., which used a population-based young-women series to assemble 87 PABC cases and 174 matched controls. While overall survival remained similar between groups, the risk of local recurrence was higher among PABC patients, particularly those diagnosed postpartum. This finding raises the possibility that local regional control may be more challenging in PABC, underscoring the importance of adequate radiotherapy and surgical margins in this setting.

#### 3.4.2. Multicenter and Registry-Based Comparative Cohorts

Larger multicenter and registry-based cohorts offer a broader view and generally involve more contemporary treatment. Amant et al. assembled an international registry of breast cancer diagnosed during pregnancy and compared 311 PABC with 865 non-pregnant controls matched for age, stage, year and country. Despite the complexity of managing cancer in pregnancy, 5-year disease-free and overall survival did not differ significantly between pregnant and non-pregnant women when treatment was delivered according to standard oncologic principles. In particular, the use of anthracycline-based chemotherapy during the second and third trimesters did not appear to compromise maternal outcomes.

Murphy et al. examined 99 women with “current or recent” pregnancy-associated breast cancer managed at a comprehensive cancer center and compared them with 186 non-PABC controls matched 2:1 on age and year of diagnosis. As in the smaller case–control series, PABC cases presented with larger, node-positive and hormone receptor-negative disease. Crude survival was inferior in the PABC group, but in multivariable models adjusting for tumor size, nodal status and receptor profile, pregnancy-associated status did not remain an independent predictor of recurrence or death.

The Korean Breast Cancer Society registry has provided several large analyses. Kim et al. identified 344 women with PABC and 688 matched non-PABC controls. PABC patients more often had high-grade, HR-negative and HER2-positive tumors, and their unadjusted survival was worse. However, after adjusting for stage, grade and subtype, the association between PABC and worse survival outcomes was not statistically significant. A subsequent registry study from Bae et al. focused on molecular subtypes in 411 PABC cases among more than 83,000 young women. Overall, PABC was associated with inferior disease-free and breast cancer-specific survival, but this effect was largely driven by luminal B and HER2-positive subtypes. Within subtype strata, survival differences narrowed, suggesting that biological phenotype rather than pregnancy per se explains much of the apparent excess risk.

Johansson et al. used the Swedish national cancer and birth registries to examine 778 women with breast cancer diagnosed during pregnancy or ≤2 years postpartum, compared with nulliparous and parous controls. Cancers diagnosed during pregnancy did not show a clear survival disadvantage after adjustment, but those diagnosed in the first two postpartum years were associated with substantially increased breast cancer-specific mortality compared with nulliparous women, even after controlling for stage and nodal status. This pattern persisted, although attenuated, when women diagnosed up to five years postpartum were considered. These results support the notion that the early postpartum period is a particularly vulnerable time period for aggressive disease behavior.

Two Korean studies found similar results for the immediate postpartum period. Gwak et al. used the national health-insurance claims data to identify 410 PABC cases and performed 1:4 propensity score matching with non-PABC controls. Overall, pregnancy-associated status was not associated with significantly different overall survival after matching. However, when PABC was stratified by timing, women diagnosed postpartum had higher mortality than their matched non-PABC controls, whereas those diagnosed during pregnancy did not. Moreover, Jo et al. conducted a prospective cohort of young breast cancer patients and similarly found that crude outcomes were worse in PABC, but the excess risk was minimized after adjusting for stage and subtype, reinforcing the central role of stage at diagnosis and tumor biology.

#### 3.4.3. Studies Focusing on Timing Within the Pregnancy–Postpartum Time Period

Several studies explicitly examined whether prognosis differed between cancers diagnosed during pregnancy and those immediately postpartum. Amant et al., Ploquin et al. and Boudy et al. all concentrated on BC during pregnancy, which was treated in specialized centers. When PABC was compared with non-PABC controls matched on conventional prognostic variables, none of these studies identified a significant survival decline associated with pregnancy. Ploquin et al. conducted a multicenter case series of early-stage breast cancer and found that 5-year disease-free and overall survival were virtually identical between pregnant and non-pregnant women once age, stage and receptor status were balanced.

On the contrary, studies that included or focused on immediate postpartum BC cases tended to show a less favorable prognosis in that subgroup. Genin et al.’s observation of higher local recurrence rates in postpartum PABC, Johansson et al.’s demonstration of increased breast cancer-specific mortality ≤ 2 year postpartum, Choi et al.’s finding that cancers diagnosed ≤1 year postpartum were associated with worse survival and the propensity-matched analysis by Gwak et al. all point in the same direction. Han et al. conducted a single-center PABC-only cohort, with no non-PABC comparator, and also found that postpartum diagnosis was associated with numerically lower disease-free and overall survival. Collectively, these studies suggested that the immediate postpartum time period may confer added risk beyond that attributable to stage and traditional prognostic factors, possibly mediated by the involuting breast microenvironment.

#### 3.4.4. PABC Series from Resource-Limited Settings

Data from low- and middle-income settings remain limited but provide important context regarding access to diagnosis and treatment. Waheed et al. represent a single-arm PABC cohort of 44 women from a tertiary hospital in Pakistan. Although it did not include a formal non-PABC comparator group, it was included to provide descriptive information on stage at diagnosis, tumor biology, treatment patterns and short-term survival in a high-burden, resource-constrained setting. No other single-arm studies meeting all methodological criteria were identified. Most women presented with large, node-positive and high-grade tumors; neoadjuvant or adjuvant chemotherapy was administered to the majority, but delays in diagnosis and treatment initiation were common. Three-year survival was lower than that reported in contemporaneous series from high-income countries, but because there was no internal non-PABC control group, it was not possible to disentangle the relative contributions of pregnancy-associated status and late-stage and health system factors. Nevertheless, the study underscores the fact that in settings where access to care is limited, the adverse features associated with PABC may be amplified by diagnostic delay and incomplete treatment.

#### 3.4.5. Overall Synthesis

Across institutional, multicenter and population-based cohorts, the weight of evidence suggests that PABC is frequently diagnosed at a more advanced stage and with more aggressive biological features than breast cancer in other young women. This translates into a worse unadjusted survival in many series. However, when modern, guideline-oriented treatment is delivered, and analyses properly account for stage, nodal status and tumor subtype, pregnancy did not appear to have a negative impact on survival rates. In contrast, multiple independent datasets indicate that breast cancer diagnosed immediately postpartum was associated with a higher risk of recurrence and death, even after adjustment for covariates. This indicates that the postpartum time period could be a distinct high-risk phenotype. These results are important for patient counselling and for the design of future studies, which should aim to investigate the role of the pregnancy–postpartum microenvironment in the prognosis of breast cancer patients.

### 3.5. Reporting Biases

Because of the substantial heterogeneity in definitions of PABC, outcomes and statistical approaches, no formal meta-analysis was performed. Therefore, conventional tools to explore publication bias, such as funnel plots or regression tests for small-study effects, were not applicable. Assessment of reporting biases was subsequently qualitative. None of the included observational studies had a prospectively registered protocol, and most did not explicitly state a priori primary outcomes. This limits the ability to judge selective outcome reporting within individual studies. Nonetheless, in almost all cohorts, the main oncologic endpoints of interest for this review, overall and disease-free or recurrence-free survival, were reported in a straightforward fashion, and there was no clear pattern of studies omitting unfavorable or unexpected results.

When comparing small single-center series with large registry-based analyses, we did not observe any systematic tendency for one group to report a more favorable impact of pregnancy-associated status. Some of the larger, better-adjusted registry studies (Kim et al., Bae et al., Johansson et al., Gwak et al. and Jo et al.) were more likely to minimize the negative impact of PABC once confounders were accounted for. On the other hand, several small institutional cohorts reported numerically worse survival outcomes for PABC, without checking for possible confounders. This pattern argues against a strong publication bias favoring “negative” or “positive” results in a single direction, although it cannot exclude more subtle selective reporting.

Our research for clinical trials or observational study registries did not identify any large, registered cohorts of PABC with survival outcomes that remained unpublished at the time of the review. However, observational prognostic research is rarely registered, and under-reporting of small institutional series with limited follow-up is plausible. Overall, while major reporting biases that would completely overturn the main conclusions seem unlikely, the lack of pre-registered protocols, the observational nature of the evidence base and the possibility of unpublished negative studies mean that selective publication and reporting cannot be ruled out and should be kept in mind when interpreting this synthesis.

### 3.6. Certainty of Evidence

We initially considered applying a structured framework, such as GRADE adapted for prognostic factor research, to formally rate the certainty of evidence for the association between pregnancy-associated status and survival outcomes. In practice, however, several features of the available data made a formal grading exercise difficult to justify and could be potentially misleading.

First, all included studies were observational and clinically heterogeneous. Designs ranged from small, single-center case–control series to large population-based registries, with substantial variation in case mix, time period and treatment availability. Second, there was no uniform definition of PABC across studies. Some cohorts restricted inclusion to cancers diagnosed during pregnancy, others combined pregnancies and cases diagnosed ≤1 year postpartum, while others extended the postpartum window to 24 months or beyond. In several reports, pregnancy and postpartum cases were pooled in a single exposure category, whereas in others, they were analyzed separately. This definitional heterogeneity, together with differences in comparator groups (nulliparous vs. parous vs. mixed non-PABC), complicated the attempt to treat all study results as a single underlying effect estimate.

Third, survival outcomes and analytic strategies were not consistent. Overall and disease-free survival were not always defined in the same way, while follow-up duration varied widely. Furthermore, the degree of adjustment for key confounders differed considerably between studies. Only a subset reported fully adjusted hazard ratios suitable for quantitative synthesis, and the covariates included in multivariable models were not standardized. Because of these discrepancies, no meta-analysis was performed. Moreover, there is no common numerical summary around which to organize a formal certainty rating.

Finally, risk of bias assessments using ROBINS-E indicated at least some concern in every study. Many institutional series were judged at high or very high risk of confounding and selection bias. Although the larger registry-based and propensity-matched cohorts provided more robust evidence and pointed in broadly similar directions, combining them with small, methodologically weaker studies into a single graded body of evidence would have led to important differences in internal validity and applicability.

For these reasons, we did not assign formal GRADE categories to the outcomes of interest. Instead, we treated the certainty of evidence as inherently limited and context dependent. We interpret the findings qualitatively, giving greater weight to large, contemporary, well-adjusted cohorts, while recognizing the constraints of the overall observational and heterogeneous evidence base.

## 4. Discussion

### 4.1. Interpretation of Findings in the Context of the Existing Literature

In this systematic narrative review of 21 observational studies, pregnancy-associated breast cancer (PABC) was consistently diagnosed at a more advanced stage and with more biologically aggressive features than breast cancer in other young women, including larger tumors, higher nodal burden, higher grade and a higher proportion of hormone receptor-negative or HER2-positive subtypes. While these features resulted in worse crude survival in many series, the majority of contemporary multicenter and registry-based cohorts demonstrated that, once analyses accounted for stage, nodal status and tumor biology, breast cancer diagnosed during pregnancy was not independently associated with poorer overall or disease-free survival. By contrast, breast cancers diagnosed in the early postpartum period consistently exhibited higher risks of recurrence and death, even after adjustment for standard prognostic factors. These findings indicate that pregnancy itself does not necessarily worsen prognosis when modern, guideline-concordant therapy is delivered, whereas early postpartum diagnosis identifies a distinct higher-risk phenotype requiring particular clinical vigilance.

This interpretation aligns only partly with earlier meta-analyses. Azim et al. combined 30 heterogeneous studies and reported that PABC was associated with a roughly 40–50% increase in mortality compared with breast cancers in non-pregnant women, even after adjustment for stage and treatment, with the strongest effect in the immediate postpartum period [33]. A more recent and larger meta-analysis by Shao et al. also concluded that PABC leads to a worse prognosis and proposed extending the PABC definition to include breast cancer diagnosis up to about six years postpartum [6]. However, these pooled estimates have merged studies with very different exposure definitions (pregnancy only, pregnancy and first postpartum year or an extended postpartum period), as well as a wide spread of time periods with various treatment protocols at the time of diagnosis. This review, which deliberately restricts the core definition of PABC to breast cancer diagnosed during pregnancy ≤ 1 year postpartum, further categorizes breast cancer diagnosis during pregnancy and postpartum.

Specifically, when pregnancy and postpartum cases are analyzed together as a single category, there is a strong risk that the poorer outcomes of postpartum breast cancer will dominate the analyses. Several methodologically stronger studies with large sample sizes in our review indicate that women diagnosed during pregnancy, treated with current oncologic standards, achieve survival outcomes comparable to non-pregnant controls once stage and subtype are taken into account in multivariable analyses (Amant et al., Kim et al., Ploquin et al. and Jo et al.). On the other hand, multiple studies concluded that breast cancer diagnosed immediately postpartum has a more aggressive behavior with poor oncological outcomes. Johansson et al. included women from the Swedish registry and showed that breast cancer-specific mortality significantly increased in women diagnosed ≤2 years postpartum, even after adjustment for stage and nodal status. Callihan et al. found a 2.8-fold higher risk of distant recurrence for cancers diagnosed within five years after delivery compared with nulliparous controls, after controlling for stage, subtype and treatment. A more recent population-based study from Shagisultanova et al. reinforced the perception that postpartum status, rather than pregnancy per se, is the main factor for poor prognosis among young women with breast cancer [34]. Our synthesis of contemporary PABC cohorts is consistent with these conclusions. When timing is analyzed, postpartum PABC repeatedly emerges as the subgroup with the highest adjusted risk for recurrence or death.

The clinical and biological plausibility of this distinction is supported by a growing body of data on postpartum breast involution. Experimental models and human tissue studies have shown that post-lactational involution is characterized by extensive extracellular matrix remodeling, immune cell infiltration and a wound-healing-like inflammatory milieu that can promote tumor invasion and metastatic dissemination [35,36,37]. Studies from Borges et al. and by Lefrère et al. further argue that the involuting breast constitutes a transient “pro-metastatic niche” that may partly explain the adverse outcomes observed for postpartum breast cancer, particularly when diagnosis occurs within the first 5–10 years after delivery [9,38]. Therefore, the results of our study, which show that pregnancy per se does not negatively affect prognosis after adequate adjustment, while diagnosis of breast cancer in the immediate postpartum period leads to poorer survival outcomes, are biologically credible.

Furthermore, our findings are generally in alignment with the conclusion of an earlier pooled analysis that “PABC” as a broad category has a worse prognosis compared to non-PABC. When PABC is defined to include BC diagnosis several years after delivery, the group includes cases with postpartum breast cancers arising in an involuting gland, leading to adverse oncological outcomes. By narrowing the PABC definition to pregnancy and ≤1 year postpartum and by examining pregnancy and postpartum diagnoses separately wherever possible, our review clarifies that this risk is not uniform across the spectrum. For clinicians, this distinction matters because it supports current guidance that pregnant women with breast cancer should be offered standard, guideline-concordant therapy without assuming a predefined worse prognosis. At the same time, our results highlight the need for hypervigilance and tailored follow-up strategies for women diagnosed immediately postpartum.

Finally, it is worth mentioning that the more favorable outcomes seen in recent multicenter and registry cohorts may also reflect improvements in systemic therapy and obstetric-oncologic coordination over time. Many of the earlier case series that were included in prior meta-analyses mainly used taxanes, trastuzumab and modern imaging, often reporting substantial therapeutic compromises during pregnancy. More contemporary cohorts, including Amant et al.’s international registry and Jo et al.’s Korean prospective series, demonstrated that when chemotherapy was delivered safely in the second and third trimesters and locoregional treatment was not unduly delayed, the survival rate for pregnant women was similar to that of non-pregnant patients. In this context, our narrative synthesis can be seen less as an overturn and more as a refinement of previous evidence by separating the influence of pregnancy, postpartum biology and historical undertreatment on the prognosis of young women facing breast cancer around the time of childbearing.

### 4.2. Limitations of the Evidence Base

The body of evidence included in this review has several important limitations that should be acknowledged when interpreting the findings. All included studies were observational. Most were retrospective case–control or cohort analyses based on hospital records or registries, with the inherent susceptibility of such designs to residual confounding and selection bias. Even in the largest datasets, adjustment for prognostic factors was incomplete and heterogeneous. Some studies controlled only for age and stage, whereas others additionally accounted for grade, receptor status, HER2, treatment and time period. None could fully account for unmeasured factors such as comorbidity, socio-economic status, diagnostic delay or subtle differences in treatment intensity, which can influence the observed associations between pregnancy status and survival outcomes.

The definition of PABC varied substantially across studies. Although we restricted our core synthesis to cohorts in which breast cancer was diagnosed during pregnancy or ≤1 year postpartum, the analyses of cases in this predefined time period varied among studies. Some authors pooled pregnancy and postpartum cases into a single exposure group, others reported them separately, while others extended the postpartum interval to 24 months or more. The timing of BC diagnosis relative to the end of pregnancy was not always clearly specified, particularly in lactation-associated cases. This definitional heterogeneity complicates direct comparison between studies and limits the precision with which prognostic differences between BC diagnosed during pregnancy and immediate postpartum cancers can be estimated.

A marked diversity in study populations and settings was observed. A considerable proportion of the evidence comes from tertiary referral centers or specialized oncology-obstetric networks, which may receive more complex cases and may not reflect the experience of smaller hospitals or community practices. Conversely, registry-based analyses often lack granular clinical detail and may under-represent very early pregnancy losses or undocumented pregnancies. Most cohorts originate from Europe and East Asia, while data from low- and middle-income countries are sparse and usually based on small, single-center series, which limits generalizability to settings where access to prompt diagnosis and comprehensive treatment is constrained.

Furthermore, survival outcome definitions and follow-up were not standardized. Disease-free and overall survival, event-free survival and recurrence-free survival were defined and measured in slightly different ways across studies. In addition, the duration of follow-up varied from just over three years in some institutional series to more than a decade in population-based cohorts, while many reports focused on early outcomes, with limited information on long-term recurrence patterns. In several older cohorts, HER2 status was unavailable for a substantial proportion of patients, meaning that trastuzumab, taxanes and other modern systemic therapies were not available during the study period, leading to heterogeneous treatment patterns.

Small sample sizes are a recurring issue in institutional case–control series; because PABC is rare, many single-center studies included fewer than 50 cases, reducing statistical power and making estimates of relative risk imprecise. Confidence intervals around hazard ratios were often wide, and subgroup analyses by timing (pregnancy vs. postpartum) or by molecular subtype were limited. In PABC-only cohorts without non-pregnant comparators, inferences about the prognostic impact of pregnancy status rely on indirect comparisons with historical or external data rather than internal controls.

Finally, reporting was variable. Few studies published a protocol or prespecified statistical analysis plan, while selective emphasis on particular models or subgroups cannot be excluded. Important details, such as the exact postpartum cut-off used, handling of missing data or criteria for including or excluding stage IV disease, were sometimes only briefly described or confined to supplementary materials. These gaps make it challenging to harmonize results across studies and further complicate any attempt to derive a single pooled effect estimate.

Overall, while the included studies provide valuable insights into the prognosis of PABC in the contemporary era, the limitations outlined above underline that these findings should be interpreted with caution. The data support broad qualitative conclusions, particularly regarding the importance of stage, tumor biology and postpartum timing, but are less well suited to precise quantification of risk or to fine-grained subgroup analyses.

### 4.3. Limitations of the Review Process

Several aspects of the review methodology itself warrant consideration. First, although the search strategy was broad and covered the major biomedical databases and trial registries, it was restricted to articles published in English from 2005 onwards. Earlier work and non-English language reports were therefore not systematically assessed and may contain additional information, particularly from regions that are under-represented in the included literature. Moreover, the grey literature and conference proceedings were not searched in a structured way, which could lead to the possibility that small or negative studies presented at meetings but never fully published were missed.

Second, despite using predefined eligibility criteria and dual independent screening and data extraction, the process remains vulnerable to human error. Abstracts and full texts were sometimes ambiguous with respect to the exact postpartum cut-off used, overlap between pregnancy and lactation categories and the inclusion of stage IV disease. Decisions about whether a study’s definition of PABC was sufficiently close to our prior definition were occasionally judgment-based. Similarly, choices about which effect estimates to extract, when several models or subgroup analyses were presented, required interpretation and may have introduced subtle selection bias.

Third, the choice to perform a narrative synthesis rather than a meta-analysis was driven by substantial heterogeneity in exposure definitions, outcomes and adjustment sets. While this approach avoids the false precision of a pooled hazard ratio based on incomparable data, it also means that the review relies on qualitative comparison, and the weight given to particular studies inevitably reflects the reviewers’ assessment of their methodological robustness. Different investigators, even with the same evidence base, might reasonably emphasize slightly different studies or interpret discordant findings in another way.

Finally, although the ROBINS-E tool provides a structured framework for evaluating bias in non-randomized exposure studies, its application to mixed cohorts that include both case–control and registry designs required some adaptation. Domain judgments, particularly for confounding and selection, are to some extent subjective. For these reasons, the risk of bias assessments should be viewed as a guide to the relative credibility of individual studies rather than as definitive rankings.

### 4.4. Implications for Practice, Policy and Future Research

From a clinical point of view, our findings reinforce and refine the message that has emerged from contemporary guidelines and expert consensus. Breast cancer diagnosed during pregnancy can be managed with curative intent using standard protocols, with appropriate adaptations for fetal safety, while maternal prognosis is not compromised compared to non-pregnant women. This supports current recommendations from ESMO, NCCN and other bodies that surgery should not be delayed and that anthracycline-based chemotherapy may be given after the first trimester at full weight-based doses, while therapeutic decisions should be primarily driven by tumor stage and subtype rather than by pregnancy alone. For everyday practice, this means that clinicians should not compromise treatment because a woman is pregnant but should carefully time systemic therapies and radiotherapy to minimize fetal exposure.

At the same time, several large cohorts and this systematic review concluded that breast cancer diagnosed immediately postpartum constitutes a higher-risk phenotype, even after controlling for stage and other prognostic factors. For clinicians, this has practical implications. When a young woman presents with breast cancer within two to five years after childbirth, it is reasonable to expect at least intermediate- to high-risk disease. Subsequently, it is important to perform a comprehensive preoperative staging and to discuss the potential for more intensive systemic therapy and close follow-up. Current breast cancer guidelines do not yet include postpartum status as a formal risk factor, but our findings and those of others suggest that incorporating postpartum timing into risk discussions and survivorship planning should be taken into consideration.

For counselling, the distinction between diagnosis during pregnancy and immediately postpartum is crucial. Young women and their partners frequently ask whether pregnancy itself has “caused” the cancer or irreversibly worsened the prognosis. Being able to explain that women treated according to guidelines during pregnancy can expect outcomes similar to non-pregnant patients can be profoundly reassuring. Conversely, for women diagnosed immediately postpartum, it is important to acknowledge the possible higher risk of recurrence and to emphasize that aggressive treatment and careful surveillance are warranted.

Our synthesis also has implications for fertility preservation and future pregnancy counselling. International guidelines from ASCO and ESMO stress the importance of timely discussion of fertility-preserving options, including oocyte/embryo cryopreservation, before gonadotoxic therapy in young breast cancer patients. The observation that appropriately treated PABC does not have a clearly worse prognosis than non-PABC supports the notion that subsequent pregnancies after completion of therapy may be considered on an individual basis. This is in line with existing data showing that pregnancy after breast cancer does not appear to increase recurrence risk [39]. However, for women whose index cancer was diagnosed immediately postpartum, clinicians should have a more cautious discussion about the timing of future pregnancies, given the higher baseline risk in this group of patients and the biological uncertainty surrounding postpartum involution.

From a policy and service organization perspective, the rarity and complexity of PABC argue strongly for centralization of care and the development of clear referral pathways to multidisciplinary teams with expertise in both oncology and high-risk obstetrics. National or regional networks and registries dedicated to cancers in pregnancy can facilitate timely access to evidence-based treatment, allow systematic capture of maternal and fetal outcomes and help standardize counselling. Routine recording of pregnancy status and timing of last childbirth in cancer registries should be encouraged. Without these data, it would be difficult to monitor incidence, outcomes and disparities in postpartum breast cancer at a population level. Particular attention is needed in low- and middle-income settings, where diagnostic delays, limited access to systemic therapy and fragmented antenatal care may compound the adverse prognosis of PABC.

Finally, our review highlights several priorities for future research. First, there is an urgent need for prospective, observational cohorts that use a standardized definition of PABC, clearly distinguishing BC diagnosis during pregnancy and postpartum and capturing, in detail, treatment, biological and outcome data. Such cohorts would allow more robust adjustment for confounding and better characterization of risk in clinically relevant subgroups, including by intrinsic subtype and germline mutation status. Second, translational studies exploring the biology of postpartum involution, including stromal remodeling, immune microenvironment changes and circulating biomarkers, should be integrated with clinical cohorts to identify potential therapeutic targets and prognostic markers. Last but not least, interventional studies, while unlikely to be randomized in the classical sense, could evaluate optimized chemotherapy schedules, supportive care and survivorship interventions specifically in pregnant and postpartum women.

In summary, our findings are consistent with evolving international guidelines. Pregnancy itself, when managed appropriately, does not necessarily preclude optimal breast cancer therapy or a realistic expectation of cure. However, breast cancer arising immediately postpartum warrants particular vigilance and is likely to remain a focus of research and policy efforts in the coming years.

## 5. Conclusions

In this systematic review, pregnancy-associated breast cancer was consistently characterized by more advanced-stage and unfavorable biological features at diagnosis compared to breast cancer in other young women. This translated into worse survival outcomes in many studies. However, in contemporary cohorts where modern, guideline-concordant therapy was delivered and analyses were adjusted for stage and tumor subtype, a BC diagnosis during pregnancy itself did not emerge as an independent adverse prognostic factor. On the contrary, breast cancer diagnosed immediately postpartum showed a higher risk of recurrence and death, even after accounting for various prognostic factors, suggesting that postpartum status identifies a distinct high-risk phenotype.

These findings support current practice recommendations that pregnant women with breast cancer should be treated with curative intent using standard protocols adapted for fetal safety and that heightened vigilance and careful follow-up are needed for women diagnosed immediately postpartum. At the same time, the evidence in the literature remains entirely observational, methodologically heterogeneous and limited by small sample sizes, particularly for postpartum subgroups. Future work should focus on prospective cohorts, with the integration of postpartum breast involution with clinical data. Improved registry capture of pregnancy and childbirth history is crucial in order to refine risk estimates and to offer precise, timing-specific guidance for women who develop PABC.

## Figures and Tables

**Table 1:** Study characteristics.

Study	Design	PABC Definition	PABC/Non-PABC (n)	Non-PABC Group
Ali 2012 [11]	Retrospective matched case–control	Breast cancer diagnosed during pregnancy or ≤12 months postpartum	40/40	Age- and stage-matched non-PABC patients from the same institution
Amant 2013 [12]	Prospective multicenter cohort with matched controls	Breast cancer diagnosed during pregnancy	311/865	Non-pregnant breast cancer controls matched for age, year, country and stage
Bae 2018 [13]	Large national registry cohort	Breast cancer diagnosed during pregnancy or ≤1 year postpartum	411/83381	All other non-PABC breast cancers in the Korean Breast Cancer Society registry
Baulies 2015 [14]	Retrospective matched case–control	Breast cancer during pregnancy or ≤1 year postpartum	56/73	Young non-PABC breast cancer patients matched for age and year of diagnosis
Boudy 2018 [15]	Retrospective case–control	Breast cancer during pregnancy or ≤1 year postpartum	49/51	Non-PABC breast cancer patients of similar age treated in same centers
Choi 2019 [16]	Retrospective cohort	Breast cancer during pregnancy or ≤1 year postpartum	63/3804	Non-PABC breast cancers in the same national database/period
Dimitrakakis 2013 [17]	Retrospective matched case–control	Breast cancer during pregnancy or ≤1 year postpartum	39/39	Non-PABC breast cancer patients matched for age, stage and treatment period
Genin 2016 [18]	Retrospective matched case–control	Breast cancer during pregnancy or ≤1 year postpartum	87/174	Non-PABC breast cancers matched 2:1 on age, year and center
Gwak 2022 [19]	Retrospective registry-based matched cohort	Breast cancer during pregnancy or ≤1 year postpartum	410/1640	Non-PABC patients matched 1:4 on age, stage and diagnosis year from same registry
Han 2020 [20]	Retrospective cohort (PABC-only)	Breast cancer diagnosed during pregnancy or ≤1 year postpartum	203/-	No formal non-PABC comparator group
Johansson 2013 [21]	Nationwide population-based cohort	Breast cancer diagnosed during pregnancy or ≤2 years postpartum (later restrict analytically to ≤1 year)	317/2965	Young non-PABC breast cancers (nulliparous and remote postpartum) in national cancer registry
Jo 2022 [22]	Prospective cohort (PABC vs. non-PABC)	Breast cancer during pregnancy or ≤1 year postpartum	93/1271	All other newly diagnosed non-PABC breast cancers in the same prospective cohort
Kim 2017 [23]	Retrospective matched case–control	Breast cancer during pregnancy or ≤1 year postpartum	344/688	Non-PABC controls matched approximately 2:1 for age and year of diagnosis
Madaras 2014 [24]	Retrospective matched case–control	Breast cancer during pregnancy or ≤1 year postpartum	31/31	Non-PABC controls matched for age, stage and institution
Martín-Cameán 2024 [25]	Single-center retrospective cohort	Breast cancer during pregnancy or ≤1 year postpartum	34/55	Young non-pregnant breast cancer patients diagnosed in same hospital and time period
Moreira 2010 [26]	Retrospective cohort	Breast cancer during pregnancy or ≤1 year postpartum	87/252	Non-PABC newly diagnosed breast cancers treated in same tertiary center
Murphy 2012 [27]	Retrospective matched cohort	Breast cancer during pregnancy or postpartum (within roughly 1 year; restricting postpartum window analytically)	99/186	Non-PABC controls of similar age and stage from same institution/period
Ploquin 2018 [28]	Retrospective matched cohort	Breast cancer during pregnancy or ≤1 year postpartum	111/253	Non-PABC matched controls (age, stage, center)
Waheed 2021 [29]	Retrospective matched case–control	Breast cancer during pregnancy or ≤1 year postpartum	44/-	No formal non-PABC comparator group
Zhang 2021 [30]	Retrospective matched case–control	Breast cancer during pregnancy or ≤1 year postpartum	58/116	Non-PABC controls matched 1:2 on age and year of diagnosis
Zhang 2023 [31]	Retrospective matched cohort	Breast cancer during pregnancy or ≤1 year postpartum	121/242	Non-PABC controls matched on age, stage and calendar period

**Table 2:** Results of individual studies.

Study Author	Year	Study Design/Data Source	PABC/Non-PABC Definition	PABC/Non-PABC (n)	Survival Outcomes (PABC vs. Non-PABC)	Other Key Findings
Ali et al. [11]	2012	Retrospective matched case–control/single tertiary center	BC diagnosed during pregnancy or ≤1 year postpartum/age- and stage-matched non-PABC controls	40/40	No significant difference in OS or DFS after matching; pregnancy status not an independent prognostic factor in multivariable models	PABC presented with larger, higher-grade tumors; ER negativity was more frequent, but differences were explained by stage and biology
Amant et al. [12]	2013	Multicenterregistry with matchednon-pregnantcontrols	BC diagnosed during pregnancy/non-pregnant young BC patients matched for age, stage and period	311/865	3–5-year DFS and OS were similar between pregnant and non-pregnant women when treated according to guidelines; pregnancy per se was not associated with worse survival.	Tumor stage and nodal status were the main determinants of outcome; chemotherapy during pregnancy was feasible without negative impact on survival
Bae et al. [13]	2018	Nationwideregistry cohort (KBCS)	BC during pregnancy or ≤1 year postpartum/all other BC in women 20–49 years	411/83,381	PABC associated with worse DFS and BC-specific survival in crude analyses; after adjustment for stage and subtype, effect was minimized, but remained adverse in luminal B/HER2-positive subgroups	PABC showed higher proportions of HR-negative, HER2-positive and high Ki-67 tumors; postpartum diagnosis was particularly enriched for aggressive subtypes.
Baulies et al. [14]	2015	Multicenter matchedcase–control	BC during pregnancy or ≤1 year postpartum/non-PABC young women matched for age, year and stage	56/73	No statistically significant difference in OS or DFS; non-significant trend towards worse DFS in PABC group	PABC tumors were larger, with higher grade and Ki-67; lymphovascular invasion was more frequent in PABC
Boudy et al. [15]	2018	Multicenterretrospectivecohort with propensity score matching	BC diagnosed during pregnancy/non-pregnant BC	49/51	After propensity score matching, 5-year DFS and OS did not differ between PABC and non-PABC	Baseline stage and biology were more adverse in PABC before matching; chemotherapy during pregnancy did not compromise maternal outcomes
Choi et al. [16]	2019	Nationwideretrospectivecohort	BC during pregnancy or ≤1 year postpartum/young women without pregnancy near diagnosis; separate group for pregnancy after BC	63/3804	BC diagnosed ≤1 year postpartum showed significantly worse BC-specific survival vs. non-PABC; cancers during pregnancy were not associated with inferior survival	Immediate postpartum BC was more often node-positive and HR-negative; postpartum interval emerged as a key prognostic time window
Dimitrakakis et al. [17]	2013	Retrospective matchedcase–control	BC during pregnancy or ≤1 year postpartum/premenopausal non-PABC matched on age, stage and year of diagnosis	39/39	Worse crude OS and DFS in PABC, but after matching and adjustment, pregnancy association was no longer a significant predictor of survival	PABC cases presented with higher stage and nodal burden; differences in outcome were largely mediated by stage at diagnosis
Genin et al. [18]	2016	Population-based matched case–control	BC during pregnancy or ≤1 year postpartum/young non-PABC from same registry	87/174	No significant difference in OS; increased risk of local recurrence in PABC, particularly when diagnosis was postpartum	PABC had more T3/T4 tumors and nodal involvement; breast-conserving surgery was more often followed by local recurrence in PABC
Gwak et al. [19]	2022	Nationwide claim-based cohort with 1:4 propensity matching	BC during pregnancy or ≤1 year postpartum/non-PABC matched on age, stage and year	410/1640	Overall PABC was not associated with significantly different OS after matching; however, postpartum PABC showed higher mortality than matched non-PABC	PABC group had more advanced-stage and aggressive subtypes before matching; postpartum interval was identified as higher-risk time period
Han et al. [20]	2020	Single-center retrospective PABC cohort	BC diagnosed during pregnancy or ≤1 year postpartum/no non-PABC group	203/-	Within PABC, postpartum diagnosis tended to be associated with worse DFS and OS vs. diagnosis during pregnancy	High prevalence of HER2-positive and TNBC subtypes and stage III disease; more aggressive phenotype in postpartum cases
Johansson et al. [21]	2018	Nationwidecancer–birthregistry cohort	BC during pregnancy or ≤2 years postpartum/nulliparous and parous women	778/1661	BC diagnosed ≤2 years postpartum was associated with substantially increased BC-specific mortality vs. nulliparous women; cancers during pregnancy were not clearly worse	Risk of death remained elevated up to several years postpartum; tumor size and nodal status only partially explained the excess risk, implicating postpartum biology
Jo et al. [22]	2022	Prospective young breast cancer cohort	BC during pregnancy or ≤1 year postpartum/other young BC in the same registry	93/1271	After adjustment for stage and molecular subtype, PABC status was not independently associated with OS or DFS; crude outcomes were worse but largely stage driven	PABC group had more advanced disease and higher rates of HER2-positive/TNBC subtypes; robust follow-up and uniform staging
Kim et al. [23]	2017	Nationwideregistry cohort with matched controls	BC during pregnancy or ≤1 year postpartum/non-PABC (1:2 matched on age and stage)	344/688	PABC was associated with worse OS and DFS; after multivariable adjustment, including stage and subtype, pregnancy association lost statistical significance	PABC tumors were more often high grade, ER/PR-negative and HER2-positive; treatment patterns were similar after matching
Madaras et al. [24]	2014	Retrospective matchedcase–control	PABC (during pregnancy or immediately postpartum)/non-PABC matched on age and stage	31/31	No significant differences in OS or DFS between PABC and non-PABC in matched analyses	PABC cases had higher stage at initial diagnosis; limited precision of survival estimates
Martín-Cameán et al. [25]	2024	Single-center retrospectivecohortwith matched controls	BC during pregnancy or ≤1 year postpartum/young nonpregnant controls	34/55	PABC associated with worse crude OS and DFS; after adjustment for stage, grade and subtype, pregnancy was not an independent predictor	PABC tumors were larger, with higher grade and HR-negative; obstetric and neonatal outcomes were favorable in PABC
Moreira et al. [26]	2010	Retrospective pairedcase–control	BC during pregnancy or ≤1 year postpartum/premenopausal non-PABC treated in same center	87/252	PABC was associated with poorer unadjusted survival; after controlling for nodal status and stage, differences in OS and DFS became non-significant	PABC group presented more frequently with node-positive and stage III disease; no evidence that pregnancy worsened prognosis after stage adjustment
Murphy et al. [27]	2012	Retrospective matched cohort	BC during pregnancy or ≤1 year postpartum/non-PABC early BC matched 2:1	99/186	PABC had inferior crude DFS and OS; in multivariable models, PABC was not an independent predictor	PABC tumors were larger, node-positive and ER/PR-negative; modern multimodality therapies mitigated prognostic disadvantage
Ploquin et al. [28]	2018	Multicenter matchedcase–control	Early-stage BC during pregnancy/non-pregnant early-stage BC matched onprognostic factors	111/333	5-year OS and DFS did not differ significantly between pregnant and non-pregnant women after matching	Careful matching on stage and receptor status suggested that, with appropriate treatment, pregnancy itself did not compromise outcomes
Waheed et al. [29]	2024	Single-center retrospective PABC cohort	BC during pregnancy or ≤1 year postpartum/no non-PABC group	44/-	3-year OS and DFS are acceptable, but somewhat lower than contemporary high-income series	Majority presented with advanced-stage and high-grade tumors; delayed diagnosis and limited resources were highlighted as major contributors to outcomes
Zhang et al. [30]	2021	Institutional matchedcase–control	BC during pregnancy or ≤1 year postpartum/non-PABC matched 1:1 on age, year, stage and subtype	41/41	No significant differences in DFS or OS between PABC and matched controls; pregnancy association was not prognostic after matching	PABC had higher rates of lymphovascular invasion and nodal positivity before matching; aggressive subtype distribution was similar after matching
Zhang et al. [31]	2023	Single-center matchedcase–control in young women	Primary BC during pregnancy, or ≤1 year postpartum/non-PABC matched 1:2 on age, stage and period	40/80	Long-term DFS and OS were comparable between PABC and non-PABC groups; no independent effect of pregnancy status was observed	PABC immediately postpartum presented with advanced-stage disease, but appropriate systemic therapy appeared to offset this disadvantage

## Data Availability

In accordance with the journal’s guidelines, the authors confirm that no new (unpublished) primary data were created or collected.

## Abbreviations

The following abbreviations are used in this manuscript:

PABC	Pregnancy-associated breast cancer
PrBC	Breast cancer diagnosed during pregnancy
PPBC	Postpartum breast cancer
BC	Breast cancer
ASCO	American Society of Clinical Oncology
ESMO	European Society of Medical Oncology
NCCN	National Comprehensive Cancer Network
ROBINS-E	Risk Of Bias In Non-randomized Studies of Exposures
HER2	Human epidermal growth factor receptor 2
DFS	Disease-free survival
OS	Overall survival
PRISMA	Preferred Reporting Items for Systematic reviews and Meta-Analyses
GRADE	Grading of Recommendations Assessment, Development and Evaluation
